# Changing Epidemiological Characteristics of Hepatitis A in Zhejiang Province, China: Increased Susceptibility in Adults

**DOI:** 10.1371/journal.pone.0153804

**Published:** 2016-04-19

**Authors:** Zhifang Wang, Yaping Chen, Shuyun Xie, Huakun Lv

**Affiliations:** Department of Immunization Programme, Zhejiang Provincial Center for Disease Control and Prevention, Hangzhou, Zhejiang Province, PR China; Universidad Nacional de la Plata, ARGENTINA

## Abstract

**Background:**

Hepatitis A is a common acute hepatitis caused by hepatitis A virus (HAV). Annually, it affects 1.4 million people worldwide. Between 1991 and 1994, HAV infections were highly endemic in Zhejiang Province (China), with 78,720 reported HAV infections per year. Hepatitis A vaccine came on the market in 1995 and was implemented for voluntary immunization. Since 2008, hepatitis A vaccine has been integrated into the national childhood routine immunization program.

**Objective:**

To understand the current epidemiological profile of hepatitis A in Zhejiang Province since hepatitis A vaccine has been available for nearly two decades.

**Methods:**

This study used the 2005–2014 National Notifiable Diseases Reporting System data to evaluate the incidence rate of notified hepatitis A cases in Zhejiang Province.

**Results:**

The overall trend of incidence rate of notified hepatitis A cases significantly decreased from 2005 to 2014 (P< 0.001). During the study period, the reported incidence rate in individuals aged ≤19 years declined to the historically lowest record in 2014. Compared with individuals aged ≤19 years, those aged ≥20 years showed the highest incidence rate (P< 0.001). Majority of HAV infected cases were Laborers, accounting for approximately 70% of reported cases.

**Conclusions:**

Childhood immunization strategy with hepatitis A vaccine seemed to be effective in decreasing notified hepatitis A incidence rate in individuals aged ≤19 years. Those aged ≥20 years were observed to be the most susceptible population. The vast majority of hepatitis A cases were notified among Laborers. Therefore, we strongly suggest that future preventive and control measures should focus more on adults, particularly Laborers, in addition to the current childhood hepatitis A vaccination programme.

## Introduction

Hepatitis A, caused by hepatitis A virus (HAV), is mostly transmitted via the fecal–oral route either by the contaminated food or water ingestion or via person-to-person contact [1]. Hepatitis A infection is usually an acute, self-limiting liver disease; however, its clinical severity is closely correlated with increasing age [2,3]. In case of children aged below 6 years, approximately 50% of children are asymptomatic; meanwhile, in children aged above 6 years and adults, 75% of infections are usually accompanied by symptoms such as jaundice and dark urine. People older than 50 years may have higher case fatality ratios (1.8%) [3–6].

Globally, symptomatic hepatitis A cases accounts for 1.4 million each year [7], with tens of millions of infections in total [8]. In China, hepatitis A is a common public health issue [9,10]. However, reported incidence rate of HAV infected cases varied across China. Historically, the average reported number of HAV infected cases in the eastern provinces of China (78,720 cases per province per year) was higher than that in the western provinces (nearly 50,000 cases per province per year) during 1990–1994 [11,12].

The above difference on geographical distribution is closely related with population density, sanitary condition, and other socioeconomic indicators [13]. Zhejiang Province is a developed eastern province in China. Compared with the provinces from western parts of China, Zhejiang Province is one of China’s smallest and most densely populated provinces, with a total area of 101,800 km^2^ and population of 54,980,000 in 2014 [14].

The most recently published study on the epidemiology of hepatitis A in Zhejiang Province was reported in 2000 [15]; according to the findings of this study, hepatitis A was highly endemic between 1991 and 1998 in Zhejiang Province, with an average number of acute hepatitis A cases per year of almost 25,000. Moreover, the highest incidence rate of hepatitis A was observed among pre-school children with a rate of >100 cases per 100,000 pre-school children.

Hepatitis A is a vaccine-preventable disease. Hepatitis A vaccines are reportedly safe and effective. In Zhejiang, these vaccines were introduced in the market in 1995. Initially, hepatitis A vaccines were available only to children whose parents were willing to voluntarily pay. Between 1996 and 2007, about 80% of hepatitis A vaccines were given to pre-school children. Since 2008, hepatitis A vaccines have been integrated in the national childhood routine immunization program, which means children aged between 18 months and 7 years are immunized free of charge. Since hepatitis A vaccines have been available for nearly two decades, it is worthwhile to reassess the evolving epidemiological profile of hepatitis A; this is critical to gather evidence for ongoing policymaking to prevent and control this disease.

In this study, we provided an update of the epidemiological characteristics of hepatitis A in Zhejiang based on population-based surveillance data collected from the National Notifiable Disease Reporting System (NNDRS) during 2005–2014. In addition, strategies for the future prevention and control of hepatitis A were discussed.

## Methods

### Surveillance of Hepatitis A in China

Since 2005, the NNDRS has been in operation in China and provides a web-based surveillance reporting platform. This surveillance platform covers the entire population living in all the prefectures, cities, and provinces. Total of 35 notified infectious diseases are electronically reportable on this platform.

Physicians or public health staff in all prefecture-, city-, and province-level hospitals or CDCs, are mandatory to manually or electronically collect data on HAV infected cases and enter them into the NNDRS on a daily basis. Notification data provided include a unique record reference number, postcode of residence, gender, age, occupation, date of onset, date of notification on the internet, and classification of a case (a suspect case or a laboratory-confirmed case). A suspect case is defined as an abrupt onset with the following symptoms: fever, headache, nausea, extreme fatigue, anorexia, vomiting, diarrhea, dark urine, clay-colored stools and jaundice. A laboratory-confirmed case is defined as any suspect case with positive anti-HAV immunoglobulin M test (in the absence of recent vaccination).

### Data resources

In this study, a dataset on all laboratory-confirmed HAV infected cases reported during January 1, 2005 to December 31, 2014 was extracted from the NNDSS. Data on population size as well as data on the study population stratified by subgroups (i.e., age and gender) were exported from statistical reports published by the Zhejiang Provincial Bureau of Statistics [14]. Additionally, data on the overall number of hepatitis A vaccines used between 2008 and 2014 were collected.

### Data analysis

Data on laboratory-confirmed hepatitis A cases were exported via the NNDSS, where non-permanent residents in Zhejiang Province and duplicated cases were removed. If one HAV infected case went to see more than one doctor within six months of the initial symptoms, this case could be reported repeatedly by different doctors. When these cases showed up in the list, they were considered as "duplicated."

Since hepatitis A vaccine was available on the market in 1995, notified incidence rate among population aged ≤19 years in 2014 may roughly represented the intervention result for the age group that has received hepatitis A vaccine. Therefore, the notification rates for those aged ≤19 years and those aged ≥20 years were analyzed separately in order to assess the effect of hepatitis A vaccine.

Occupations were chosen from the Chinese Standard Classification of Occupations 1995: (1) Farmers, fishermen, and laborers who perform physical tasks; (2) Sales and service workers; (3) Leading cadres; (4) Police officers, health personnel, and teachers; and (5) Others. In this study, we abbreviated these as five respective classes: (1) Laborers; (2) Workers; (3) Managers; (4) Professionals; and (5) Others.

To calculate the incidence rate, the number of notified hepatitis A cases during 2005–2014 were divided by the Zhejiang Provincial Census population for each matching year. Incidence rate was calculated per 100,000 population. The rates, stratified by the overall population, gender, and age group were adjusted to the age distribution of the 2014 Zhejiang standard population using the direct method described [16,17]. Chi-square test was used to investigate the relationship between hepatitis A incidence rate and different variables (for example, age and gender). One-way analysis of variance (ANOVA) was used to assess significant differences among the means of age. Linear regressive method was performed to analyze trends in incidence rates by year or doses of vaccine used by year. Data were analyzed using Statistical Product and Service Solutions (SPSS, Inc., Chicago, IL, USA, version 22.0), and Microsoft Excel 2013. *P*< 0.05 indicated statistical significance.

## Results

From January 1, 2005 to December 31, 2014, a total of 18 duplicated records were removed. Consequently, 8923 laboratory-confirmed cases were reported to the NNDRS in Zhejiang Province (Range: 423–1696 cases per year; Mean:892.3 cases per year) during the study period. The annual incidence rate was 1.7 cases per 100,000 population (Range: 0.8–3.4 cases per 100,000 population). The mean age of reported HAV cases was 38.8 years.

### Overall trend of hepatitis A incidence rate

**Fig 1:**
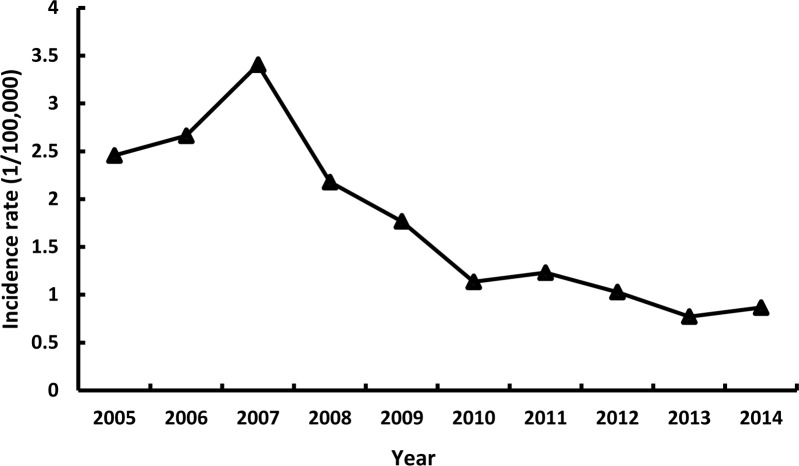
Hepatitis A incidence rate by year.

Linear regression analysis revealed that a decreasing trend of HAV reported incidence rate in Zhejiang Province during the study period (P<0.001), although the trend was observed to be upward during 2005–2007 and then be downward between 2008 and 2014 (Fig 1).

### Age distribution

**Fig 2:**
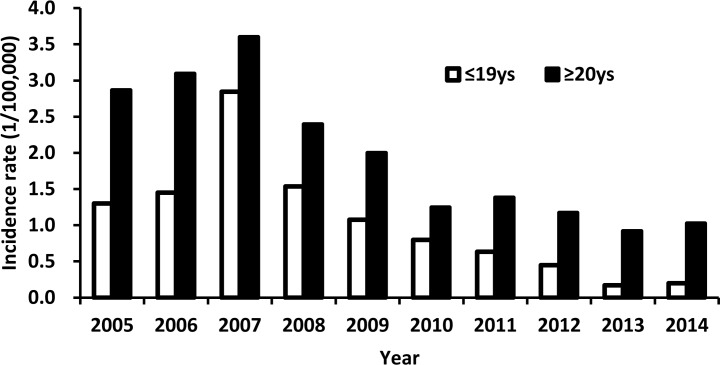
Hepatitis A incidence rate by age group and year.

During 2005–2007, the incidence rate increased in individuals aged ≤19 years and those aged ≥20 years (*P*< 0.001, Fig 2). However, between 2008 and 2014, the incidence rate declined in the two age groups (*P*< 0.001, Fig 2). The most drastic decrease was observed among those aged ≤19 years; the incidence rate sharply decreased by 87.0%, which represented a decline from 1.68 cases per 100,000 individuals in 2008 to 0.22 cases per 100,000 individuals in 2014.

The mean age of reported hepatitis A cases shifted by >10 years during the study period, with a right shift from 36.8 years in 2005 to 47.2 years in 2014 (*P*< 0.001).

### Gender distribution

**Fig 3:**
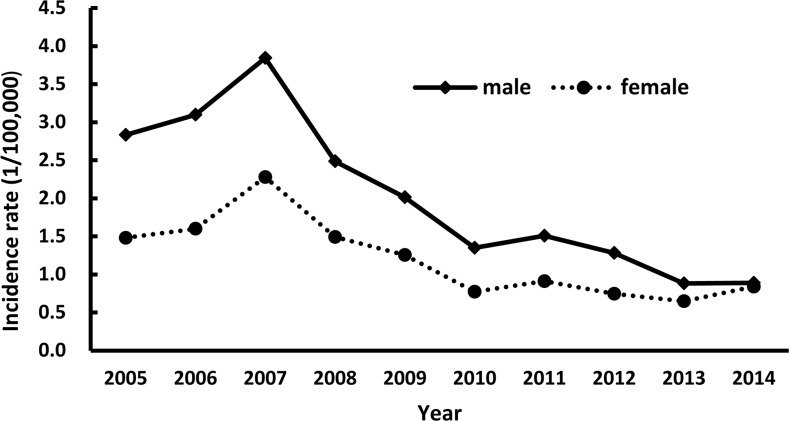
Hepatitis A incidence rate by gender and year.

During the study period, the incidence rate of reported hepatitis A cases were higher in males than in females (*P*< 0.05, Fig 3). The male-to-female ratio was 2:1 during 2005–2007; the gap slowly narrowed from 1.8:1 in 2008 to 1.1:1 in 2014.

### Occupational distribution

The hepatitis A cases in 2005–2014 were reported among population from different occupations (Table 1). Every year, laborers made up the vast majority of HAV cases during the study period. The proportion of laborers appeared to tower over the proportions of other occupations: Laborers, 68.8%; Professionals, 5.1%; Workers, 4.2%; Managers, 3.2%; and Others, 18.7%.

**Table 1:** Percentages of hepatitis A cases by occupation.

Year	L	P	W	M	O	Total
2005	74.5	4.9	4.1	2.3	14.1	100.0
2006	75.6	3.6	2.9	1.6	16.3	100.0
2007	67.1	3.4	2.7	3.2	23.6	100.0
2008	67.0	4.4	3.4	2.9	22.4	100.0
2009	72.6	3.6	3.0	3.4	17.3	100.0
2010	65.0	6.5	4.4	3.6	20.6	100.0
2011	64.0	6.4	5.5	3.3	20.7	100.0
2012	62.3	8.0	4.8	4.8	20.1	100.0
2013	66.0	5.4	7.8	6.1	14.7	100.0
2014	60.7	12.6	12.4	4.4	9.9	100.0
2005–2014	68.8	5.1	4.2	3.2	18.7	100.0

L: Laborers; W:Workers; P:Professionals; M:Managers; O:Others.

### Hepatitis A vaccines used

Due to a limited number of vaccines available in 2008, one dose of live attenuated hepatitis A vaccine was initially used for 18–24-month children in a few cities which had a higher prevalence of HAV. Subsequently, throughout Zhejiang Province, hepatitis A vaccines began to be given to children 18 months to 7 years of age by 2010. During 2005–2014, there was no a specific catch-up vaccination program implemented to any certain age cohort in Zhejiang Province.

From 2008 to 2014, the number of hepatitis A vaccines used was noted to continuously increase from 100,000 doses in 2008 to 800,000 doses in 2014 (*P*< 0.001, Table 2). From 2011 to 2014, the average number of vaccines used each year (700,000 doses) appeared to be sufficient for one child birth cohort (500,000–600,000 children).

**Table 2:** Dose of Hepatitis A vaccines used and new-born population by year.

Year	Dose used	New-born population
2008	100,000	510,000
2009	300,000	510,000
2010	400,000	520,000
2011	700,000	540,000
2012	700,000	550,000
2013	800,000	550,000
2014	800,000	550,000
Total	3,800,000	3,700,000

## Discussion

This study describes a changing trend in the incidence rate of reported HAV infections that occurred before and after massive introduction of hepatitis A vaccine. Before the national childhood routine vaccination program was introduced in 2008, the overall incidence rate continuously increased from 2.5 cases per 100,000 individuals in 2005 to 3.4 cases per 100,000 individuals in 2007. Nevertheless, a significant decline in the incidence rate was noted from 2.2 cases per 100,000 individuals in 2008 to 0.9 cases per 100,000 individuals in 2014. These upward and downward trends could be explained by the following two factors. First, before introducing childhood vaccination program, hepatitis A was endemic and characterized by cyclical periods of approximately every 7–10 years [18–20]. Previously, in Zhejiang Province, a higher prevalence of hepatitis A infection was seen in 1960, 1967, 1981, 1988, and 1996 [15,21]. Therefore, the slightly higher reported incidence rate in 2007 is presumably related to the cyclical epidemiology; a few outbreaks occurred in primary schools in this province as a result of drinking contaminated water [22,23]. Second, a large number of doses of hepatitis A vaccines administered to children each year is believed to be responsible for this steady decline, and this is in agreement with data published by a previous study [24].

During 2013–2014, the annual average incidence rate of reported HAV infections declined to the historically lowest record of <1.0 case per 100,000 individuals; this rate was similar to that in developed countries with low endemicity, such as the United States of America (0.4–0.6 per 100,000 population between 2009–2013) [25], Germany (1.0/100,000 in 2012) [26], Denmark (1.0/100,000 in 2012) [26], and the Netherlands (0.7/100,000 in 2012) [26]. Zhejiang Province may therefore be classified as a low-endemic area. This hypothesis needs further support from studies on the seroprevalence of antibodies to HAV among the general population.

Our results showed that the reported incidence rates for each age group declined between 2008 and 2014, although only children aged ≤19 years appeared to benefit directly from the province-wide routine vaccination programs. The decline in the hepatitis A incidence rate is closely correlated with the level of socioeconomic development and improvement in living conditions [27,28]. With urbanization policies being extensively implemented throughout China, more people are relocating from rural to urban areas. During 2007–2012, in Zhejiang Province, >50% of the rural adult population had significantly improved their living conditions and sanitary infrastructures by migrating [29]. In addition, the booming economy and changing lifestyles contribute to disrupting the fecal–oral transmission of HAV, thereby reducing the likelihood of getting infected [30].

The difference in incidence rate by gender occurred during the study period, with a higher incidence rate in males than in females, which is consistent with the findings of other studies [31–33]. There is no reported biological evidence to support that males are more susceptible to HAV infection than females. The difference may be explained by gender disparities with regard to access to healthcare resources. Usually, females tend to have poorer access to healthcare resources than males due to their comparably limited opportunity for education and paid labor.

The present study also showed that majority of the reported HAV infections were seen among the Worker class; this finding conspicuously differed from the national research study results obtained in 2005–2007 [1]. The current study identified adults with a low socioeconomic status to be the most susceptible population, whereas the national research study identified peasants and children of <10 years as the most susceptible populations [1]. This difference may be explained by the provincial inequality of economic development. In western, underdeveloped provinces in China, HAV transmission predominately occurs among children, whereas in eastern, developed provinces, HAV transmission primarily occurs among adults. The mean age of infection was noted to shift by 10 years in the present study, which depicted an increased risk with regard to the potential severity of infection in the aging population. Therefore, we strongly recommend that adults with a low socioeconomic status should be the focus of hepatitis A immunization, along with the continued implementation of the current country-wide routine vaccination program in children.

## Conclusions

This study reflected the change of epidemiological features of HAV infection between 2005 and 2014 in Zhejiang Province. The mean age of infected HAV cases shifted from 36.8 to 47.2 years. The reported incidence rate in individuals aged ≤19 years declined to the historically lowest record in 2014. The vast majority of reported hepatitis A cases were observed in the Worker class. Our findings highlight the importance of future public health efforts that focus on protecting the Worker class from hepatitis A.

Hepatitis A infection risk is closely related with the poor sanitation, lack of safe drinking water, consumption of contaminated food with HAV, and underdeveloped economic areas [34–38]. Therefore, improved sanitation, safe food and hepatitis A vaccines are considered to be the most efficient ways to combat this disease. In addition, future research are needed to explore the sources of exposure to HAV for the susceptible population and determine the level of HAV exposure. On the basis of the current massive routine vaccination program, we suggest additional measures are needed to prevent and control HAV infections among the population at risk, such as, health education for improved personal hygiene practices, providing sustainable access to safe drinking water or food, and improved sanitation for living space.

## Supporting Information

S1 File Dataset on reported hepatitis A cases during 2005–2014. (SAV)
